# CT as a Diagnostic Tool for Emphysematous Pyelitis: A Case Report

**DOI:** 10.7759/cureus.13513

**Published:** 2021-02-23

**Authors:** Pranav Ajmera, Sruthi Krishnamurthy, Manish Joshi, Laveena Ajmera

**Affiliations:** 1 Radiology, Dr. D.Y. Patil Medical College, Hospital and Research Center, Pune, IND; 2 Emergency Medicine, Dr. Ram Manohar Lohia Hospital, New Delhi, IND; 3 Radiology, Satara Hospital & Research Center Private Limited, Satara, IND; 4 Radiology, Dr. Ajmera Diagnostic, New Delhi, IND

**Keywords:** pneumoureter, pneumobladder, pneumopelvis, emphysematous pyelonephritis, emphysematous pyelitis

## Abstract

Gas forming infections of the renal collecting system occurs because of organisms like Escherichia coli, Klebsiella, and Proteus. If the gas is restricted to the collecting system, without causing involvement of the cortex, it is called emphysematous pyelitis; whereas, invasion and penetration of the cortex imply a more gruesome diagnosis of emphysematous pyelonephritis.

A 59-year-old male patient, previously diagnosed with a large right renal calculus and having multiple co-morbidities presented to the surgery department with right flank pain; Double J (DJ) stenting was done to relieve the pain from colic due to obstructive renal calculi; the patient subsequently discharged without any post-procedural complications. The patient came back a month later with similar complaints and multiple spikes of fever. Blood and urine culture revealed growth of Escherichia coli. The first line radiological investigations, like X-ray and ultrasonography, were suggestive of the presence of air in the pelvis, ureter, bladder; confirmation by CT revealed the presence of air in the collecting system, including the calyx. This air was seen invading focally into the anterior renal cortex. Also, the DJ stent had migrated into the proximal ureter.

The patient had developed emphysematous pyelitis predominantly, which had developed an overlapping component of pyelonephritis. The aetiology for air in the renal system was infection by Escherichia coli. CT proved to be diagnostic in differentiating both of them, as the presence of air entering the renal cortex was detected only on CT. Subsequently, prominent initiation of antibiotic therapy and replacement of DJ stent was carried out, following which the patient recovered fully within two weeks.

## Introduction

Gas forming infections of the renal system, vary in their severity, depending on the area involved. If the infection involves the renal parenchyma, it invariably has higher mortality as compared to the isolated involvement of the renal collecting system. These are more common in those with diabetes as a co-morbidity or some aetiology causing long-term obstruction of the urinary pathway [1]. The organisms generally responsible for gas formation include Escherichia coli, Klebsiella, Proteus, and Pseudomonas [2]. Herein, we discuss such a case of emphysematous pyelitis, complicated by a large renal calculus. However, the case also had some overlapping features of focal emphysematous pyelonephritis.

## Case presentation

A 59-year-old male patient presented to the medical department with complaints of diffuse, continuous pain over the right flank, for a few weeks. It was associated with spikes of fever. His associated co-morbidities included diabetes mellitus type-2 and coronary artery disease. The patient did not have any relevant family history. On evaluation, he was diagnosed with an obstructive right renal calculus with smaller ureteric calculi causing hydro-ureteral-nephrosis, and subsequently, a Double J (DJ) stent was put on the right side. Lithotripsy could not be carried out as the patient had recently undergone angioplasty; he was on regular dual anti-platelet therapy and hence was deemed unfit for operation. He was admitted for a week post-intervention and was advised to come back after three months, for lithotripsy. However, a month later, there was an increase in pain on the right side along with complaints of burning micturition, urgency, nocturia for nearly a week, and episodes of fever. The physical examination of the patient was unremarkable. The patient’s blood Investigations at this time revealed deranged renal function tests (RFTs), with serum urea=108 mg/dL and serum creatinine=3.8mg/dL, serum glucose levels=270 mg/dL (random). His complete blood counts were mildly elevated at 13,500/μl and neutrophil count at 63%. Subsequently, urine and blood culture were sent. Urine samples returned growth of Escherichia coli on culture and microscopy revealed 4-5 pus cells/high power field. Blood culture too revealed the presence of Escherichia coli.

**Figure 1:**
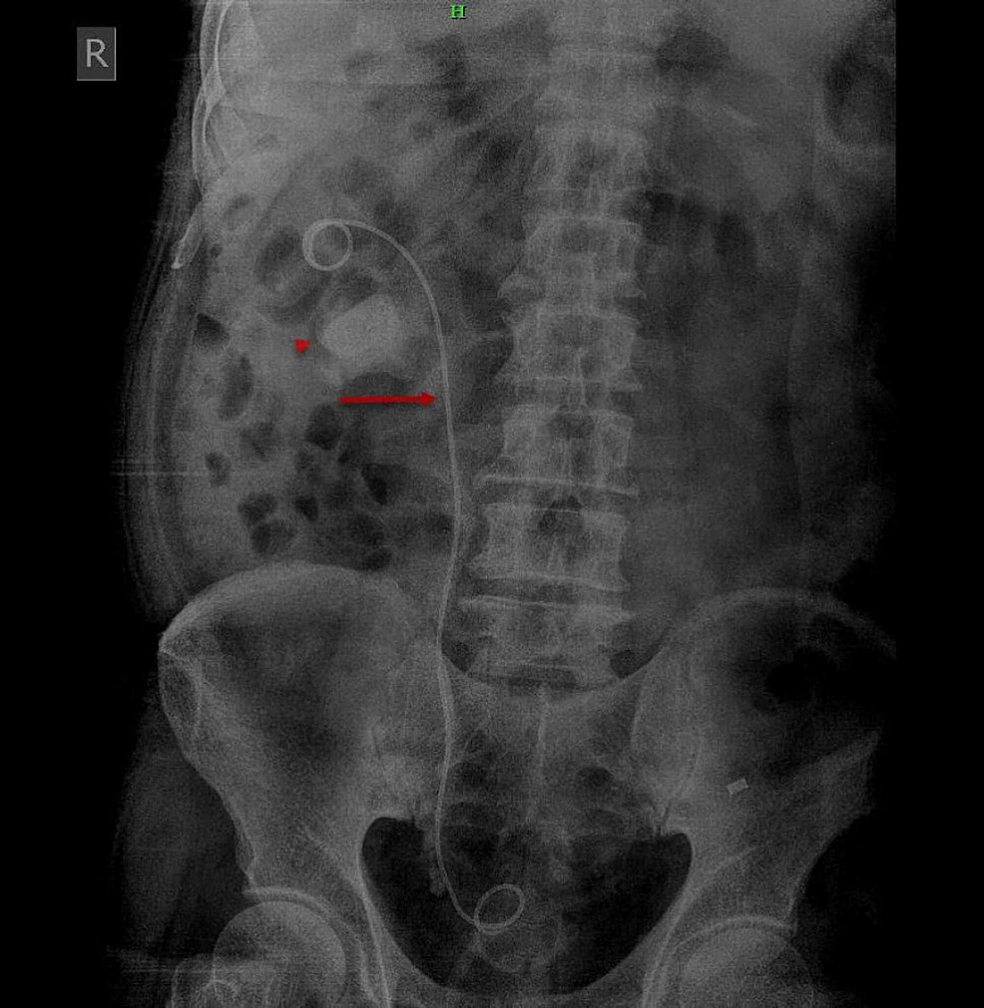
X-ray of kidney-ureter-bladder revealed a DJ stent on the right side (red arrows). A large radiodense calculus was noted on the right side (red arrowhead).

The patient was referred to the radiology department for an X-ray kidney-ureter-bladder (KUB), which revealed a DJ stent on the right side with a large right renal calculus at the level of the L2 vertebra. A suspicious abnormal collection of gas was noted in the right paravertebral region overlying the region of the right ureter but it was difficult to differentiate it from intra-bowel gases (Figure 1).

Subsequently, the patient underwent ultrasonography (US) of the abdomen and pelvis with a curvilinear transducer, which revealed a large right renal calculus with multiple smaller lower ureteric calculi associated with right renal hydro-ureteral-nephrosis. Internal echoes with extensive shadowing were noted within the right renal pelvicalyceal system. Based on these findings, a presumptive diagnosis of right pyonephrosis was made.

**Figure 2:**
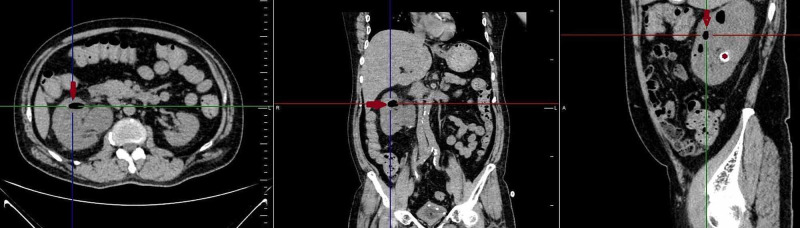
Reformatted plain CT abdomen in axial, coronal, and sagittal sections revealed air in the renal pelvis and calyces (red arrows). A large radiodense calculus (red asterisk) was seen towards the inter-polar region on the sagittal section.

In view of the above findings, the subsequent day the patient underwent a plain CT-KUB, as contrast-enhanced-CT could not be performed due to the deranged RFTs. Through the night, the patient's pain in the right flank had increased and become nearly continuous. The CT revealed the presence of a large, radiodense calculus of size nearly 20 (cranio-caudal) x 21 (antero-posterior) x 19 (transverse) mm in the lower pole of the right kidney associated with right-sided hydronephrosis and smaller renal calculi in the lower ureter causing hydroureter. Multiple air pockets were noted in the right pelvicalyceal system, predominantly in the upper and middle calyces (Figure 2).

**Figure 3:**
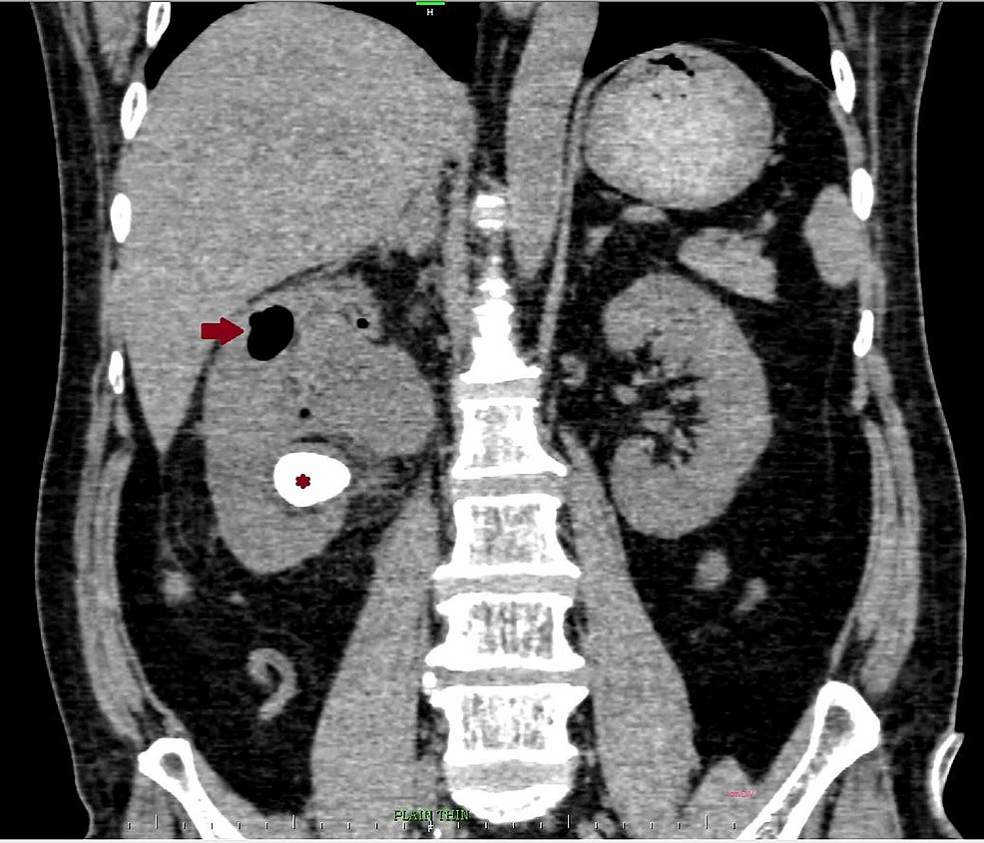
Sagittal reconstruction of CT abdomen revealed a large air pocket (red arrow) near the superior pole of the kidney. The air pocket was seen extending as a smaller pocket into the renal cortex, that was giving rise to emphysematous pyelonephritis. A large renal calculus was noted extending towards the lower pole (red asterisk).

Few small air foci were seen in the anterior cortex of the right kidney (Figure 3).

**Figure 4:**
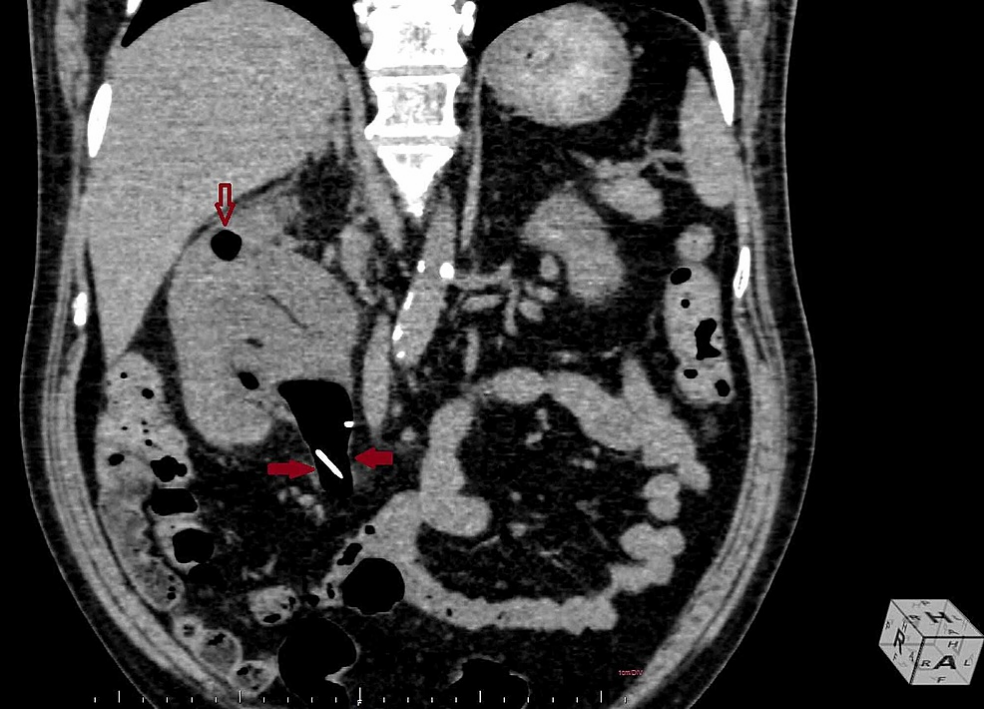
Sagittal reconstruction of CT abdomen revealed a massive air pocket in the pelvis of right kidney, extending into the upper ureter (red arrows). The proximal end of the DJ stent was noted in the upper ureter, suggestive of migrated DJ stent. A small air pocket was noted in the upper calyx, indenting into the renal cortex (empty red arrow).

These findings were in favour of emphysematous pyelitis with mild changes of emphysematous pyelonephritis. Air pockets were noted in the dependent portion of the right ureter, suggestive of right pneumoureter (Figure 4).

**Figure 5:**
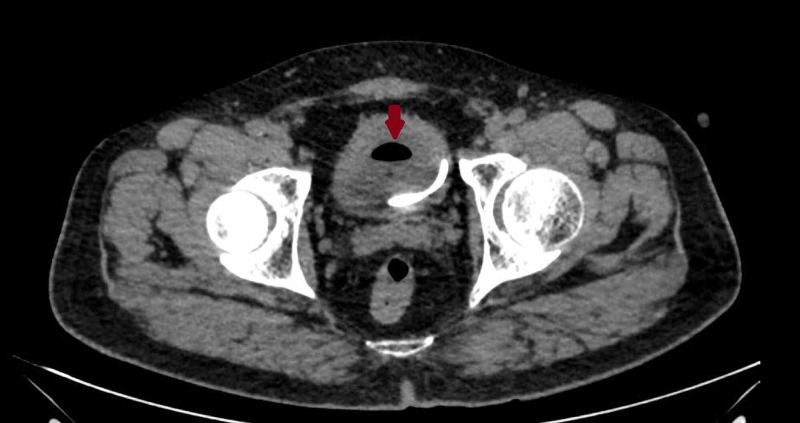
Axial section of CT abdomen revealed the presence of air in the bladder (red arrow) and the distal end of the DJ stent.

DJ stent on the right side had migrated distally with the proximal end lying just distal to the right pelvic-ureteric junction. The urinary bladder was noted to be partially distended with foleys bulb-in situ and had a large air pocket within, suggestive of a pneumobladder (Figure 5).

At no point could we visualise any fistulous connection with the gastrointestinal system, neither did the patient give any such history to suspect the same. The patient’s DJ stent was replaced with another one and the patient was kept on conservative antibiotic therapy with ciprofloxacin for two weeks. Post antibiotic urine and blood culture revealed no growth.

## Discussion

Emphysematous pyelonephritis is a potentially life-threatening infection of the upper urinary tract with gas-forming organisms that have gas formation within or surrounding the renal parenchyma. Other possible differentials of gas in the renal parenchyma include trauma, iatrogenic, entero-renal fistula. It is classified into four classes by Huang and Tseng whereby, some label class-1 emphysematous pyelonephritis itself as emphysematous pyelitis. Both have a strong association with diabetes mellitus. However, pyelitis almost always develops secondary to obstruction [2-4].

Emphysematous pyelitis is a condition characterised by gas in the renal collecting system and has a lesser risk and conservative management as compared to pyelonephritis [2]. In our case, the air was mostly present in the renal collecting system, causing pneumoureter and pneumobladder. However, at a focal location on the right side, it seemed to be slightly invading the anterior renal cortex.

The diagnosis of emphysematous pyelitis is often mistaken clinically as emphysematous pyelonephritis, due to the significant overlap in symptomatology. Both present with flank pain, fever, and chills [1]. However, it is very important to be able to differentiate between the two, as the management protocols for both differ. Gas in renal parenchyma requires drainage or nephrectomy while pyelitis can be managed with conservative antibiotic therapy and correction of the obstruction, if possible [1,2,5-7].

USG findings serve as pointers towards the diagnosis, but it is not possible to accurately differentiate air, calculus, and calcifications [1]. CT is the imaging modality of choice, in diagnosing and following-up gas containing infections of the urinary tract. It is possible to give an accurate diagnosis along with an accurate staging on CT [1,3,4].

We can safely rule out the cause for air in the ureter to not be procedural (post-DJ stenting), as then we would have visualised the presence of a significant amount of air at the time of discharge after the procedure. Due to the obstructive hydronephrosis and consequent urine stasis, the site was suitable for infective bacteria to breed. Subsequently, extensive gas production was carried out by Escherichia coli. 

## Conclusions

In conclusion, this patient while predominantly suffering from emphysematous pyelitis also had features of focal emphysematous pyelonephritis. CT, by demonstrating the subtle parenchymal invasion which could not be picked on the US, proved to be the diagnostic modality in arriving at the timely diagnosis, as otherwise, the pyelitis component would have continued to increase, causing increased renal parenchymal damage and an increase in morbidity. The patient improved symptomatically within a week of initiation of antibiotic therapy; pharmacological therapy lasted for two weeks.

## References

[1] Sallami S (2010). Emphysematous pyelitis complicated by renal calculi: a case report. UroToday Int J.

[2] Sureka B, Thukral BB (2012). Emphysematous infections of the urinary tract: a radiological perspective. Indian J Nephrol.

[3] Weng WC, Pu YS, Yu HJ, Huang CY (2012). Pneumopelvis, pneumoureter and pneumobladder. QJM.

[4] Kua CH, Abdul Aziz Y (2008). Air in the kidney: between emphysematous pyelitis and pyelonephritis. Biomed Imaging Interv J.

[5] Hiremath R, Mahesh Mahesh, Padala KP, Swamy K, Pailoor A (2015). A rare case of pneumoureter: emphysematous pyelitis versus emphysematous pyelonephritis. J Clin Diagn Res.

[6] Chiang CC, Jong YS, Wang WJ (2010). Emphysematous pyelitis. CMAJ.

[7] Pontin AR, Barnes RD (2009). Current management of emphysematous pyelonephritis. Nat Rev Urol.

